# Protocol for testing global neuronal workspace and integrated information theories of consciousness in non-human primates and mice

**DOI:** 10.1371/journal.pone.0342770

**Published:** 2026-02-25

**Authors:** Matilda Gibbons, Ethan G. McBride, Raghuram Holenarasipura Venkatasubbaiah, Majid Khalili-Ardali, Jacqulyn R. Kuyat, Samuel Gale, Vayle LaFehr, Nathan W. Vogler, Mohammadali Kheirkhah Ravandi, Ming-Jiun Tsai, Corbett Bennett, Arjun Sridhar, Benjamin Hardcastle, Hannah Cabasco, Christof Koch, Lionel Naccache, Giulio Tononi, Shawn Olsen, Maria Neimark Geffen, Theofanis I. Panagiotaropoulos, Yuri B. Saalmann

**Affiliations:** 1 Department of Otorhinolaryngology, University of Pennsylvania, Philadelphia, Pennsylvania, United States of America; 2 Allen Institute, Seattle, Washington, United States of America; 3 Department of Psychology, University of Wisconsin – Madison, Madison, Wisconsin, United States of America; 4 Cognitive Neuroimaging Unit, CEA, INSERM, Université Paris-Saclay, NeuroSpin Center, France; 5 Tiny Blue Dot Foundation, Santa Monica, California, United States of America; 6 Paris Brain Institute, Sorbonne University, Paris, France; 7 Department of Psychiatry, University of Wisconsin – Madison, Madison, Wisconsin, United States of America; 8 Department of Psychology, National and Kapodistrian University of Athens, Athens, Greece; 9 Centre for Basic Research, Biomedical Research Foundation of the Academy of Athens (BRFAA), Athens, Greece; 10 Wisconsin National Primate Research Center, Madison, Wisconsin, United States of America; Public Library of Science, UNITED STATES OF AMERICA

## Abstract

Identifying the neural correlates of consciousness (NCC) is widely regarded as imperative to our understanding of consciousness. Although several scientific theories of consciousness make predictions about the NCC, they often differ in their level of detail, making direct comparison through experimental findings difficult. Here, we report an adversarial collaboration protocol in which unbiased experimentalists will test predictions formed by theory proponents of Global Neuronal Workspace Theory (GNWT) and Integrated Information Theory (IIT), concerning the location, timing and functional connectivity of the NCC. To enable cross-species comparisons, non-human primates (NHPs) and mice will view/listen to supra-threshold visual/auditory stimuli for variable durations in a go-nogo task that controls for report confounds, while we record with Neuropixels electrodes neuronal responses from visual, auditory, posterior parietal and/or prefrontal cortical areas. To causally test predictions about the timing and location, we will manipulate activity in prefrontal cortical regions using electrical stimulation in NHPs or optogenetic silencing in mice. This Study Protocol details the experimental design, analyses, divergent predictions, and the anticipated outcomes, along with their interpretation.

## Introduction

The problem of relating subjective conscious experiences to the objective, physical brain is often described as the “Hard Problem” of consciousness [1]*.* It is not only an intellectual problem of the first order, but also one that carries major scientific, clinical, legal, ethical, and societal implications, particularly in determining who or what is conscious: an unresponsive ICU patient? A second-trimester fetus? A lobster? A cortical organoid? An artificial system such as a large language model?

For the past thirty years, a key focus of the consciousness science community has been to identify the neural correlates of consciousness (NCC), the minimal neuronal mechanisms jointly sufficient for any one conscious experience, and to differentiate these from the enabling factors necessary for consciousness *tout court*, such as appropriate metabolic, hemodynamic and neuromodulatory factors [2,3].

In parallel, scientific theories of consciousness have emerged, with various amounts of empirical support [4]. Yet these theories often have very different metaphysical commitments with flagrantly opposing predictions concerning the NCC. In an attempt to adjudicate, the Templeton World Charity Foundation established five adversarial collaborations, designed to directly pit predictions of two theories against each other [5] using best practices in open science: large sample sizes, replication across theory-neutral labs, pre-registration, and open access to all data and analysis code.

The first adversarial collaboration, named Cogitate, tested predictions from two theories that come to different conclusions with respect to the NCC in humans [6,7], Integrated Information Theory (IIT) [8,9] and Global Neuronal Workspace Theory (GNWT) [10,11]. IIT proposes that the quality of an experience corresponds to a system’s cause-effect structure (or Φ structure) – composed by the set of causes and effects specified by different parts of the system on each other, and their overlap [12]. IIT uses a mathematical framework to quantify the amount of integrated information specified by this structure with a measure called Φ. Through examining the composition and quantity of the Φ structure, IIT suggests that we can understand the quality and quantity of any one conscious experience, respectively. A key limitation of IIT raised by critics is that, currently, Φ cannot be computed for a system as complex as the human brain [13] (but also see: [14]). GNWT, in contrast, posits that consciousness arises when selected information is globally broadcast across the brain, creating a ‘global workspace’ [10], allowing otherwise nonconscious processing pathways to access this information which enables integration and rerouting of information. GNWT suggests that, when new information enters the workspace, a sudden, global ‘ignition’ signal occurs, representing the moment of conscious access. A key limitation of GNWT raised by critics is the lack of empirical data regarding the nature of ignition at the level of neurons [15].

Although not all aspects of these two theories are mutually exclusive [16], there are some testable hypotheses that are directly comparable and opposable. These hypotheses broadly relate to the location (Question 1), timing (Question 2) and functional connectivity (Question 3) of the NCC. Regarding the location (Table 1, Q1), IIT predicts that the NCC are found in regions that maximize cause-effect powers. Given the lattice-like, “topographic” connectivity of visual, auditory and tactile cortical areas, which likely underlies maximal irreducibility, the neural substrate of consciousness and the various NCC should be found primarily in posterior cortical regions, including sensory cortical areas [17]. Conversely, GNWT predicts that the NCC are found in the higher order associative areas, in particular the prefrontal cortical regions (PFC) and posterior parietal cortical regions (PPC) [18,19], but also in other areas that are accessible via top-down mobilization. As for the timing of the NCC (Table 1, Q2), IIT predicts that their timing is sustained in the posterior cortex for as long as the stimulus is consciously perceived, because the quality of any one experience should be identical to the particular composition of the cause-effect structure, itself specified by the system in a particular state. GNWT, however, predicts brief “ignitions” at the onset of conscious perception, followed by the active and sustained maintenance of the accessed representation within the global workspace, as long as the individual remains conscious of it. Ignition at offset is not a direct prediction of GNWT because it is predicted to occur only if the participant is aware of this offset [20]. Finally, regarding the functional connectivity of the NCC (Table 1, Q3), IIT predicts that posterior cortical regions that specify the conscious contents that bind to create the experience of a particular stimulus should be linked by short-range synchrony, because causal relations (bindings) are predicted to depend on an overlap in inputs and outputs, and that shared inputs and outputs of activated areas should translate into increased phase-synchrony during the experience of the stimulus. GNWT, however, predicts long-range synchrony between distant areas, because information between distant regions should be globally integrated to create a conscious experience.

**Table 1:** Prediction table outlining the analysis plan for theory-specific predictions of consciousness.

Question	Dataset			Hypothesis	Sampling plan	Analysis plan	Interpretation given to different outcomes	Theory interpretation	
	Response variable	Sample	Time window					GNWT	IIT
**Question 1a: Where** is conscious content represented?	Average decoder performance	PFC	Stimulus onset	**H**_**0**_: Performance is equal to chance level **H**_**1**_: Performance is greater than chance level	Data will be collected until the Bayes factor is at least 10X in favor of H_1_ over H_0_	Half Cauchy prior for H_1_	**A.** Bayes < 1/10: Strong evidence for H_0_ *or* **B.** Bayes factor > 10: Strong evidence for H_1_	**Pass:** Requires B **and** D	**Pass:** Requires F
		PPC		**H**_**0**_: Performance is equal to chance level **H**_**1**_: Performance is greater than chance level	Data will be collected until the Bayes factor is at least 10X in favor of H_1_ over H_0_	Half Cauchy prior for H_1_	**C.** Bayes < 1/10: Strong evidence for H_0_ *or* **D.** Bayes factor > 10: Strong evidence for H_1_		
		PFC; PPC; sensory regions	Stimulus duration	**H**_**0**_: Performance combining PPC and/or sensory regions is equal to that in PFC **H**_**1**_: Performance combining PPC and/or sensory regions is greater than in PFC	Data will be collected until the Bayes factor is at least 10X in favor of H_1_ over H_0_	Half Cauchy prior for H_1_	**E.** Bayes < 1/10: Strong evidence for H_0_ *or* **F.** Bayes factor > 10: Strong evidence for H_1_		
**Question 1b:** Is PFC required for consciousness?	Average decoder performance	PPC, w/ PFC manipulation	Stimulus onset	**H**_**0**_: No difference in performance in PPC with PFC manipulation **H**_**1**_: Performance in PPC changes with PFC manipulation (for NHPs) **H**_**2**_: Performance in PPC is lower with PFC manipulation (mice)	Data will be collected until the Bayes factor is at least 10X in favor of either H_1_ or H_2_ over H_0_	Full Cauchy prior for H_1_; half Cauchy prior for H_2_	**A.** Bayes < 1/10 for H_0_ vs. H_1_: Strong evidence for H_0_ *or* **B.** Bayes >10 for H_0_ vs. H_1_: Strong evidence for H_1_ **C.** Bayes < 1/10 for H_0_ vs. H_2_: Strong evidence for H_0_ *or* **D.** Bayes >10 for H_0_ vs. H_2_: Strong evidence for H_2_	**Pass:** Requires B for NHPs **and** D for mice	**Pass:** Requires F
		PPC, sensory regions, w/ PFC manipulation		**H**_**0**_: Performance combining PPC and/or sensory regions is equal to chance level **H**_**1**_: Performance combining PPC and/or sensory regions is greater than chance level	Data will be collected until the Bayes factor is at least 10X in favor of H_1_ over H_0_	Half Cauchy prior for H_1_	**E.** Bayes < 1/10 for H_0_ vs. H_1_: Strong evidence for H_0_ *or* **F.** Bayes >10 for H_0_ vs. H_1_: Strong evidence for H_1_		
**Question 2.** When is conscious content represented?	Average decoder generalization	PFC	Stimulus onset	**H**_**0**_: Generalization is equal to chance level **H**_**1**_: Generalization is greater than chance level	Data will be collected until the Bayes factor is at least 10X in favor of H_1_ over H_0_	Half Cauchy prior for H_1_	**A.** Bayes < 1/10 for H_0_ vs. H_1_: Strong evidence for H_0_ *or* **B.** Bayes >10 for H_0_ vs. H_1_: Strong evidence for H_1_	**Pass:** Requires B and D; weak theoretical commitment to F and H.	**Pass:** Requires J
		PPC		**H**_**0**_: Generalization is equal to chance level **H**_**1**_: Generalization is greater chance level			**C.** Bayes < 1/10 for H_0_ vs. H_1_: Strong evidence for H_0_ *or* **D.** Bayes >10 for H_0_ vs. H_1_: Strong evidence for H_1_		
	Average spike rate	PFC	Stimulus offset	**H**_**0**_: Spike rate is equal to baseline **H**_**1**_: Spike rate is greater than baseline	Data will be collected until the Bayes factor is at least 10X in favor of H_1_ over H_0_	Half Cauchy prior for H_1_	**E.** Bayes < 1/10 for H_0_ vs. H_1_: Strong evidence for H_0_ *or* **F.** Bayes >10 for H_0_ vs. H_1_: Strong evidence for H_1_		
		PPC		**H**_**0**_: Spike rate is equal to baseline **H**_**1**_: Spike rate is greater than baseline			**G.** Bayes < 1/10 for H_0_ vs. H_1_: Strong evidence for H_0_ *or* **H.** Bayes >10 for H_0_ vs. H_1_: Strong evidence for H_1_		
	Average decoder generalization	PPC and/or sensory regions	Stimulus duration	**H**_**0**_: Generalization is equal to chance level **H**_**1**_: Generalization is greater than chance level	Data will be collected until the Bayes factor is at least 10X in favor of H_1_ over H_0_	Half Cauchy prior for H_1_	**I.** Bayes < 1/10 for H_0_ vs. H_1_: Strong evidence for H_0_ *or* **J.** Bayes >10 for H_0_ vs. H_1_: Strong evidence for H_1_		
**Question 3.** What functional connectivity supports consciousness?	Pairwise phase consistency	PFC with PPC	Stimulus onset	**H**_**0**_: Pairwise phase consistency is equal to baseline **H**_**1**_: Pairwise phase consistency is greater than baseline	Data will be collected until the Bayes factor is at least 10X in favor of H_1_ over H_0_	Half Cauchy prior for H_1_	**A.** Bayes < 1/10 for H_0_ vs. H_1_: Strong evidence for H_0_ *or* **B.** Bayes >10 for H_0_ vs. H_1_: Strong evidence for H_1_	**Pass:** Requires B, D and F	**Pass:** Requires H and J for NHPs **and** J for mice
		PFC with sensory regions		**H**_**0**_: Pairwise phase consistency is equal to baseline **H**_**1**_: Pairwise phase consistency is greater than baseline	Data will be collected until the Bayes factor is at least 10X in favor of H_1_ over H_0_	Half Cauchy prior for H_1_	**C.** Bayes < 1/10 for H_0_ vs. H_1_: Strong evidence for H_0_ *or* **D.** Bayes >10 for H_0_ vs. H_1_: Strong evidence for H_1_		
		PPC with sensory regions		**H**_**0**_: Pairwise phase consistency is equal to baseline **H**_**1**_: Pairwise phase consistency is greater than baseline	Data will be collected until the Bayes factor is at least 10X in favor of H_1_ over H_0_	Half Cauchy prior for H_1_	**E.** Bayes < 1/10 for H_0_ vs. H_1_: Strong evidence for H_0_ *or* **F.** Bayes >10 for H_0_ vs. H_1_: Strong evidence for H_1_		
		Higher-order with lower-order sensory regions	Stimulus duration	**H**_**0**_: Pairwise phase consistency is equal to baseline **H**_**1**_: Pairwise phase consistency is greater than baseline	Data will be collected until the Bayes factor is at least 10X in favor of H_1_ over H_0_	Half Cauchy prior for H_1_	**G.** Bayes < 1/10 for H_0_ vs. H_1_: Strong evidence for H_0_ *or* **H.** Bayes >10 for H_0_ vs. H_1_: Strong evidence for H_1_		
		Within sensory regions		**H**_**0**_: Pairwise phase consistency increase is equal to baseline **H**_**1**_: Pairwise phase consistency increase is greater than baseline	Data will be collected until the Bayes factor is at least 10X in favor of H_1_ over H_0_	Half Cauchy prior for H_1_	**I.** Bayes < 1/10 for H_0_ vs. H_1_: Strong evidence for H_0_ *or* **J.** Bayes >10 for H_0_ vs. H_1_: Strong evidence for H_1_		

This table details the analysis plan for testing the predictions of the Global Neuronal Workspace Theory (GNWT) and Integrated Information Theory (IIT) across the three questions regarding conscious content: 1) Where is it represented?, 2) When is it represented?, and 3) What functional connectivity supports it? Each row specifies the null and alternative hypotheses, the relevant brain region(s), the time window of interest, sampling plan, the response variable used (average decoding performance or generalization, or pairwise phase consistency), and the interpretation given to the possible outcomes both in terms of theory-dependent requirements and theory-independent conclusions. Bayesian inference is used throughout, with hypotheses evaluated based on the strength of the Bayes Factor (BF). A BF > 10 in favor of H_1_ or H_2_ over H_0_ is interpreted as strong evidence for the alternative hypothesis, and a BF < 1/10 as strong evidence for the null. Each outcome is labeled (A–J) and corresponds to the schematic visualizations in Fig 3.

Cogitate tested these predictions in 250 subjects tested on tasks that required consciousness and recorded with fMRI, EEG, MEG and implanted ECoG electrodes, using several decoding analyses [6]. Their results both supported and challenged both theories. For Question 1, their results supported IIT overall, as conscious content was robustly represented in the PPC and sensory regions, and only partly in the PFC, but note that the absence of a conscious versus unconscious contrast prevented the isolation of strict NCC in that study. For Question 2, they found that neural activity was sustained throughout the conscious perception in posterior areas and there was no ignition signal at stimulus offset, supporting IIT and challenging GNWT. Finally, for Question 3, they found no evidence for sustained short-range synchrony in the PPC and sensory regions, and partially some long-range dynamic functional connectivity between the PFC and sensory areas, supporting GNWT.

Our adversarial collaboration builds directly on this framework, extending it to two additional mammalian species: rhesus macaques (*Macaca mulatta)* and mice (*Mus musculus*). This approach offers two major advantages: 1) direct access to the activity of individual neurons with intracranial electrophysiology, and 2) the ability to causally manipulate brain activity using electrical stimulation and optogenetics. The former has the advantage of directly measuring neuronal population spiking activity, thus providing substantially higher spatiotemporal resolution compared to methods like fMRI, EEG or ECoG. The latter specifically means that we can answer Question 1 *causally*, by directly manipulating activity in the PFC and assessing whether activity and stimulus decodability is modulated in the PPC and sensory regions (GNWT prediction) or persists without being obliterated in the PPC and/or sensory regions (IIT prediction).

**Fig 1:**
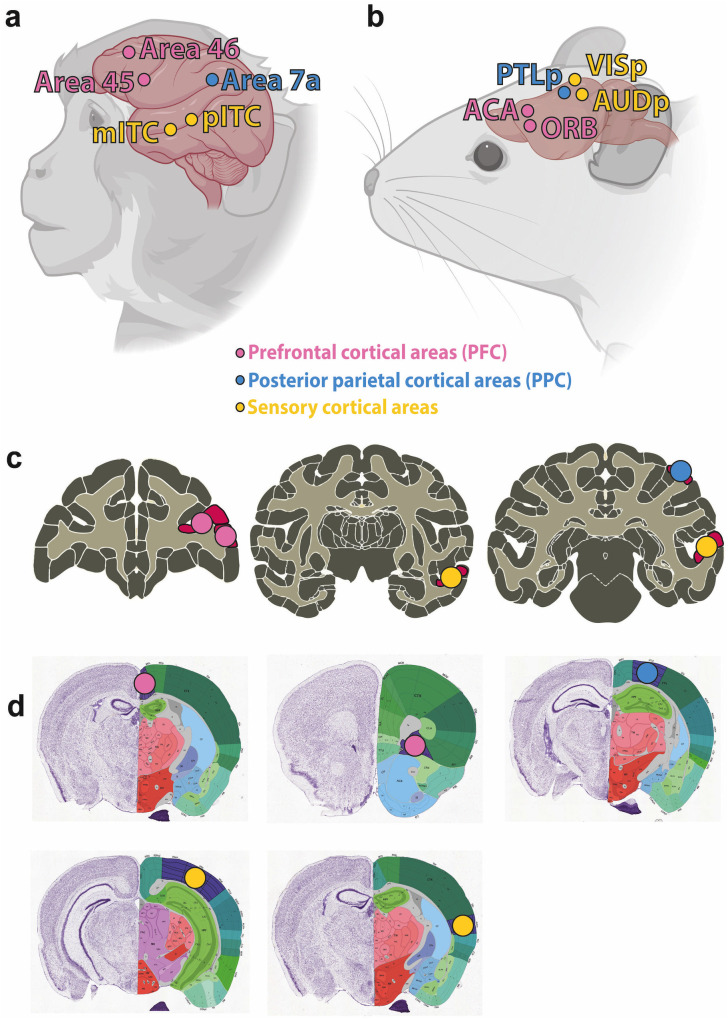
Comparative anatomical location of recording sites across species. This figure illustrates the anatomical regions used in our decoding and connectivity analyses in non-human primates (NHPs) **(a, c)** and mice **(b, d)**. Prefrontal cortical areas (PFC) are depicted by pink circles, posterior parietal cortical areas (PPC) by blue circles, and yellow circles represent sensory regions. All locations are schematic and approximate. **(a)** Approximate illustration of the recording sites in NHPs and **(b)** in mice. **(c)** Corresponding coronal sections containing the recording sites in NHPs from the Scalable Brain Atlas [26,27] and **(d)** in mice from the Allen Mouse Brain Atlas [28,29]. **(a, b)** Created in BioRender. Gibbons, M. (2026) https://BioRender.com/mdbdh1b. Reprinted under a CC BY license, with permission from BioRender, original copyright 2026.

We designed a similar set of experiments to Cogitate, adapted for non-human primates (NHPs) and mice. Neuronal activity will be recorded using Neuropixels probes in multiple areas of the NHP or mouse cortex (Fig 1) while subjects perform a go/no-go discrimination task involving supra-threshold stimuli, presented one at a time, and for a long duration. Task-irrelevant stimuli are interleaved with targets in order to minimize behavioral report confounds. These stimuli, including the task-irrelevant stimuli, are assumed to be consciously perceived–given their salience, duration, and lack of competing stimuli–an assumption that is supported by prior evidence of consciousness in both macaques [21,22] and mice [23,24]. The task is designed to allow for testing different aspects of neuronal responses to the stimuli to differentiate between predictions of the two theories [25].

Assessing the predictions for Question 1 (location) will involve testing whether PFC, PPC and/or sensory region activity can be used to decode task-irrelevant and/or non-target stimuli (specific theory predictions are outlined in the analysis plan and Table 1). Additionally, we will manipulate PFC activity on a subset of trials to test whether the PFC is required for stimulus decodability in the PPC and/or sensory regions. To test Question 2 (timing), stimulus decoding in these areas will be measured using a moving temporal window across the length of the stimulus presentation. We will test Question 3 (connectivity) by analyzing content-specific spike-LFP phase synchrony within and between PFC, PPC and sensory regions, testing for content-specific long- or short-range synchrony.

Importantly, the predictions tested here are not unique to GNWT or IIT; other prominent theories of consciousness also suggest similar neural mechanisms. For instance, higher-order theories implicate the PFC, while re-entry theories emphasize the role of the PPC and sensory regions [4,30]. As such, we expect our findings will be inherently valuable for advancing the broader search for the NCC, regardless of whether they align more closely with one theory or another. Accordingly, we do not treat our results as definitive proof for or against any specific theory. Rather, we interpret them as evidence about the neural basis of consciousness, evidence that may support or challenge many theories.

We test predictions in both macaques and mice to take advantage of the different experimental strengths of each species. Macaques have more similar neuroanatomical organization to humans and allow for more complex behavioral paradigms; whereas viral and genetic techniques are more advanced in mice, allowing for greater control over causal manipulations. Considering that the latency of neural responses in cortical areas is generally shorter in mice, due to, e.g., their smaller brain size, it is possible that the timing of the perceptual mechanisms in question may be earlier in mice than primates. So we will test the hypothesis (outside of theory hypotheses) that stimulus information is represented earlier in each area in mice compared with macaques [31]. To do this, we will decode stimuli using a moving temporal window across the length of the stimulus presentation. This allows us to examine the degree to which mechanisms are shared across species, providing insight into the evolution of the NCC.

Together, this work aims to provide a rigorous, cross-species test of competing models of consciousness, using both observational and causal tools to determine where, when, and how conscious experience arises in the brain.

## Materials and methods

### Ethics information

The research procedures comply with all relevant ethical regulations and adhere to the *National Institutes of Health Guide for the Care and Use of Laboratory Animals*. NHP experimental protocols have been approved by the Institutional Animal Care and Use Committee (IACUC) at the University of Wisconsin–Madison. Mouse experiment protocols have been approved by the IACUCs at the Allen Institute and the University of Pennsylvania. This research follows the ARRIVE guidelines [32] for reporting animal studies.

### Animals

NHP experiments will be conducted in adult male rhesus macaques (*Macaca mulatta*; age 8–9 years; weight 10–15 kg). Animals will be socially-housed in a temperature-controlled environment (22–26 °C) under a 12-hour light/dark cycle, and will receive daily fresh produce alongside standard primate chow. Water intake will be regulated according to behavioral training protocols. All experiments will be conducted during the macaques’ active (light) phase, in full compliance with institutional care standards. Animals are under the care of full-time veterinarians, veterinary technicians, and the general care staff in addition to the research team. The research team and the behavioral management unit carry out an environmental enrichment program to ensure the psychological health of the monkeys and to prevent environmental stress. Toys and other interactive devices are provided on a rotational basis and new toys are introduced regularly. The animals also see a member of the research team or animal care staff each day and are provided with primate treats during these interactions. The behavioral training relies on positive reinforcement (juice, raisin and nut rewards).

Mouse experiments will be conducted in adult male and female *Mus musculus*, either C57BL/6 (Stock No. 000664), Vgat-ires-Cre (Slc32a1; Stock No. 028862), or Ai32 × Vgat-ires-Cre (Stock Nos. 024109, 028862) lines, aged 60–250 days and weighing 18–30g (The Jackson Laboratory). Mice will be single-housed in a temperature-controlled environment (28 °C) under a 12-hour light/dark cycle. Food will be available *ad libitum*, and water will be restricted in accordance with behavioral protocols. All experiments will be conducted during the animals’ dark (active) cycle. In a subset of mice, we will conduct auditory brainstem response measurements using standard methods [33] to ensure that the mice are not undergoing premature hearing loss.

### Overall design

Subjects will perform a discrimination task in which they are presented with a supra-threshold stimulus, either an image of a face or object (NHPs) or an auditory tone or visual grating (mice), that may be task-relevant or irrelevant. These two modalities were used in mice because of their limited capacity to discriminate among multiple categories of complex visual stimuli. During the task, we will record both single unit spiking activity and local field potentials (LFPs) using high-density Neuropixels silicon probes targeting five cortical regions. In a subset of trials, activity in PFC will be manipulated: electrically stimulated using a linear electrode array in NHPs [34,35], or optogenetically silenced in mice via activation of channelrhodopsin-expressing inhibitory neurons using an optical fiber or focused laser [36,37].

Our primary analysis, similar to the approach used in the Cogitate human study, will involve decoding spiking activity related to sensory content, under the assumption that subjects consciously perceive the supra-threshold stimuli. Data collection and analysis will not be blinded to experimental conditions. However, we will randomize stimulus presentation, inter-trial intervals, and manipulation conditions (electrical stimulation vs. no stimulation in NHPs; optogenetic silencing vs. no silencing in mice).

### Brain region terminology

We use the terminology in Table 2 to refer to subsets of brain regions targeted for electrophysiological recordings.

**Table 2:** Brain area terminology in non-human primates (NHPs) and mice.

Brain regions	*NHPs*	*Mice*
**Prefrontal cortical regions (PFC)**	Area 45 (including the face-related area prefrontal ventral [38]) and Area 46 (prefrontal arcuate)	Anterior cingulate area (ACA) and orbital frontal cortex (ORB)
**Posterior parietal cortical regions (PPC)**	Area 7a	Posterior parietal association area (PTLp)
**Sensory Areas**	Posterior inferior temporal cortex (pITC), including the posterior lateral face patch (PL), and middle inferior temporal cortex (mITC), including the middle lateral face patch (ML), as well as object-selective areas	Primary visual cortex (VISp) and primary auditory cortex (AUDp)

This table displays the targeted cortical areas in NHPs alongside their functionally-similar homologs in mice across three regions: prefrontal cortical regions (PFC), posterior parietal cortical regions (PPC), and sensory areas.

### Behavior

**Fig 2:**
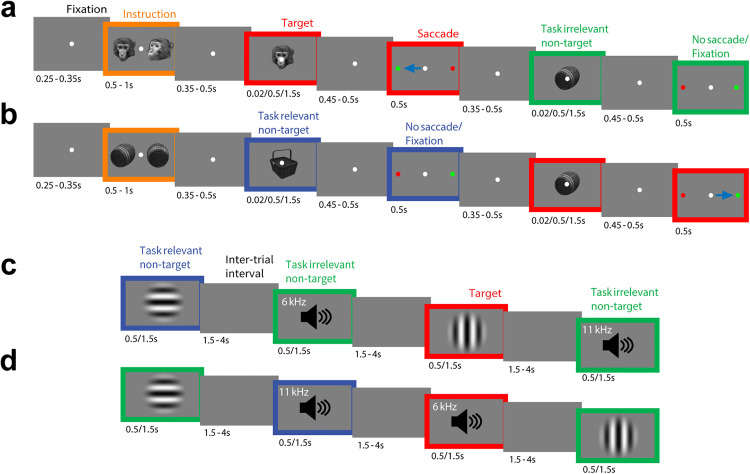
Experimental paradigm. Behavioral task structure for NHP **(a)** face and **(b)** object target trials, and for mouse **(c)** visual and **(d)** auditory target trials. **(a, b)** Cascade of screenshots showing initial cue instructing the NHP about the target stimulus in the trial—face in **(a)** and object in **(b)**—followed by two stimuli in random order. The stimuli will be the target (e.g., face in **a)**, non-target (a different face) or irrelevant stimulus (object in **a)**. Stimuli will be presented one at a time for a duration of either 0.02, 0.5 or 1.5 s (chosen randomly). On presentation of a target stimulus, the NHP will saccade (blue arrow) to a peripheral circle (green) to report the target (side and thus saccade direction will be randomized). After making a saccade, fixation is reacquired, and the next stimulus will be presented. NHPs will maintain central fixation for the non-target and irrelevant stimuli. 0–2 targets will be presented per trial. The peripheral circle location and saccade direction will be varied randomly across trials. On one-third of 0.02 s trials, the stimulus (e.g., face) will be quickly followed by a mask (scrambled face); on another third of 0.02 s trials, the stimulus will be omitted and just the mask will be presented; on another third of 0.02 s trials, only the stimulus will be presented. **(c, d)** One group of mice will be trained that visual stimuli are targets **(c)**, and a balanced group will be trained that auditory stimuli are targets **(d)**. When visual stimuli are targets, the auditory stimuli will be the irrelevant stimuli, and vice versa. Stimuli will be presented one at a time for a duration of 0.5 or 1.5 s (chosen randomly). Each trial will consist of a random pre-stimulus period (1.5-4 s; mean 2 s) during which the screen is gray, followed by a stimulus and response period (3 s; includes 0.5 or 1.5 s stimuli). For the last 1.5 seconds of the pre-stimulus period, the mouse must withhold licking, otherwise a new random pre-stimulus period is drawn and the trial restarts. Four trials are shown for each example session. The speaker icon is shown for illustration purposes only, during auditory trials the visual display is uniform gray.

Both NHPs and mice will perform a go-nogo behavioral task in which they must discriminate target stimuli from non-target relevant stimuli and from task-irrelevant stimuli (Fig 2).

### Set-up

NHPs will sit upright in a primate chair (Crist Instruments, Hagerstown MD), with their heads stabilized using a head holder (Neuronitek, London, Canada). NHPs will be positioned 57 cm from a 19 inch CRT monitor (100 Hz refresh rate). NHPs will use eye movements to perform the behavioral task and earn juice rewards. Eye position will be monitored using a video eye tracker (SMI, Berlin, Germany). Behavioral task events, monitoring of eye position and feedback delivery will be controlled by custom-written software in Presentation (Neurobehavioral Systems, Albany CA). Juice rewards will be delivered via a TTL pulse issued to an infusion pump system (Harvard Apparatus, Holliston MA).

Mice will be head-fixed on a freely-moving wheel with a lick spout that delivers 5 μL water rewards extended in front of them. There will be a screen facing the mouse’s right eye and a speaker above and to the side of the mouse. We will also use an infrared camera and light source to record the size of the mouse’s pupil that is viewing the screen.

### Task design

One category (or modality) of stimuli, either faces or objects for NHPs and visual or auditory stimuli for mice, will be task relevant while the other category will be task irrelevant. Which modality is task relevant will change for each trial in the NHP task and for each individual subject in the mouse version (counterbalanced across mice).

### Stimuli

In the NHP task, the targets will be both the front and side views of the particular instructed face or object and the non-targets will be any other face or object. The visual stimuli will be presented in grayscale at 100% luminance contrast, with a visual angle of 6 × 4 degrees. The face stimuli will be obtained using the PrimFace Database [39], and the object stimuli from the Object Databank [40].

For mice, the targets will be either a vertically-oriented grating or a 6 kHz pure tone, and the non-targets will be a horizontal grating or an 11 kHz pure tone. The visual stimuli will be presented in grayscale at 100% luminance contrast, with a spatial frequency of 0.04 cycles/degree and a size of 50 degrees. Auditory stimuli will be presented at 65 dB sound pressure level relative to 20 µPa.

### Task

For NHPs, we will aim to present 160 targets (40 face front, 40 face side, 40 object front, 40 object side), 160 non-targets and 320 task-irrelevant stimuli in each recording session (exact numbers will depend on the NHP behavior in any given session). For mice, we will aim to present 150 targets (150 vertical gratings or 150 6 kHz tones), 150 non-targets (150 horizontal gratings or 150 11 kHz tones), and 300 task-irrelevant stimuli (150 of either both visual stimuli or both auditory stimuli) in each recording session. Each stimulus will have a duration of, for NHPs, either 0.5 or 1.5 s (or 0.02 s for masked trials), followed by a delay of 0.45–0.5 s, and, for mice, either 0.5 or 1.5 s followed by a delay of 1.5–4 s (drawn from an exponential distribution). The order of target, non-target and irrelevant stimuli presentation will be pseudorandomised.

For NHPs, the trials will start with fixation on a central white circle for 0.25–0.35 s, then an instruction cue will appear for 0.5–1 s to signal the target stimulus for the duration of the trial. They will then experience a variable delay of 0.35–0.5 s before being presented with the first test stimulus. After a further delay of 0.45–0.5 s, two peripheral, colored circles, one green and one red, will appear on either side of the central fixation. The peripheral circles and the correct saccade direction will be randomly varied from trial-to-trial, to minimize action preparation during test stimulus presentation. After the response, the NHPs will be required to re-engage central fixation to initiate the second test stimulus presentation. 0–2 targets will be presented per trial. The time between test stimuli will depend on reaction time and the re-engagement of central fixation. As an awareness control (not present in the Cogitate human study), we will incorporate a backward masking manipulation into a subset of the trials with 0.02 s stimulus duration. On one-third of these masking trials, the stimulus will be followed by a mask (scrambled face) of 0.04 s duration; on another third of trials, the stimulus will be omitted and just the mask will be presented; in addition to the one-third of trials in which only the stimulus will be presented.

Mice will start the session with five trials of the target stimulus paired with a water reward to promote participation. For the rest of the session, target, non-target stimuli and irrelevant stimuli will be pseudorandomly presented to the mouse. Mice will be required to withhold licking during a 1.5 s quiescent period where the screen is blank (gray) just prior to each stimulus, otherwise the trial will restart with a randomly-chosen pre-stimulus time between 1.5–4 seconds (exponential distribution).

### Reporting of target stimuli

NHPs will report the target stimulus by a saccadic response away from a central white circle they are fixated on to a peripheral green colored circle, and not saccading to the distractor peripheral red circle on the other side of the screen (sides will be randomly varied from trial-to-trial). This response will be rewarded by administering a juice reward at the end of the trial, but only if the correct response is shown to both test stimuli. The correct response to non-targets and irrelevant stimuli will be to maintain central fixation on the white circle. Incorrect responses to the non-target and irrelevant stimuli will not be punished. To control for possible saccade-related neuronal activity, we will also collect neuronal data in a guided saccade task to the two locations of the peripheral circles, at the start of each session.

Mice will report the target stimulus by licking the water spout within the response window (0.1–1 s following stimulus onset). This response will be rewarded by administering a water reward immediately. The correct response to non-targets and irrelevant stimuli will be to withhold licking. Incorrect responses to the non-target and irrelevant stimuli will not be punished.

### Training

We will employ a stepwise training protocol to familiarize each head-fixed NHP with color-coded peripheral circles, which serve as response cues. In the initial phase, each NHP will be trained to maintain central fixation for progressively longer durations. Once stable fixation performance is achieved, a white peripheral circle will be introduced, prompting the NHP to execute a saccade toward its location. Subsequently, green and red peripheral circles will be presented, with the green circle serving as a cue for saccading to its location and the red circle serving as a distractor. Each trial will begin with an instruction cue, followed by the presentation of a test stimulus (relevant target, non-target, or irrelevant). The NHP will be trained to saccade toward and fixate on the green peripheral circle when a target stimulus is presented, while maintaining central fixation on the white circle when a relevant non-target or irrelevant stimulus is presented. The location of the green and red peripheral circles, as well as the saccade direction, will be randomized across trials. Initial training will involve presenting a single test stimulus per trial. Once the NHP demonstrates consistent and accurate performance (~85% accuracy), a second test stimulus will be introduced. In this phase, after responding to the first stimulus, the NHP will be required to re-establish central fixation to initiate the presentation of the second test stimulus. The test stimuli will vary in duration (0.5 or 1.5 seconds). Correct responses to both test stimuli will be reinforced with a juice reward at the end of the trial, while incorrect responses to either stimulus will not be rewarded. The NHP must maintain stable task performance with an overall accuracy of approximately 85% before proceeding to neuronal recordings.

Mice will be habituated by handling for five minutes each day for five days. They will then be water restricted to 85% of their baseline weight. Following this, mice will be trained to lick from the water spout by being given manually-dispensed water rewards, freely at first and then only when the mouse licks. Then, mice will be trained to lick when shown the target stimulus by presenting the mice with 150 randomly-interleaved target and non-target stimuli, with free water rewards alongside the target stimulus, and the quiescent period will be introduced. When mice lick for at least 75% trials in a session, they will move to the main training stage. The main training procedure will introduce parts of the behavioral task structure, including the initial five autorewarded targets, the pseudorandom stimulus presentation and the criteria for reward and punishment. However, it differs from the full behavioral task in a few ways. First, if a mouse fails to respond to the target after ten consecutive target trials, then an autoreward will be dispensed on the 11th presentation of the go stimulus to promote participation. Second, an incorrect response to the non-target stimuli will be punished with a 3 s timeout paired with an error cue stimulus (a black screen for visual target mice and white noise for auditory target mice). Third, all stimulus durations will be 0.5 seconds. Finally, the task-irrelevant stimuli will not be presented. When mice achieve a d-prime of 2 for two out of three consecutive days on this task, they will move on to the full behavioral task, in which irrelevant stimuli will be introduced, stimuli will vary in duration (0.5 or 1.5 seconds), and incorrect responses to non-target or irrelevant stimuli will not be punished. Mice must then maintain a d-prime of 2 for two days on the behavioral task before undergoing the electrophysiology experiment.

### Surgery

To access the intended brain recording sites, all animals will undergo a head post surgery, to allow for head stabilization during experiments, and a craniotomy procedure, to allow for insertion of the probes. All surgeries will be performed under general anesthesia, maintained using either isoflurane gas (1–3% isofluorane/O_2_) for mice and NHPs (using an endotracheal tube), or via a constant rate infusion of propofol (0.1–0.6 mg/kg/min, IV) for NHPs only. All surgeries will be performed under sterile conditions with the animal secured in a stereotaxic frame.

In NHPs, the head post surgery will be performed prior to behavioral training, and will involve attaching an acrylic head implant, head post and recording chamber to the skull. This procedure will start with preparing the scalp through hair removal and antiseptic cleansing, then making an incision along the midline to expose the skull. Then, using a hand drill with a safety cuff to limit drill descent (cuff size will be based on the individual’s skull thickness determined from presurgical structural MRI), 12 threaded holes (2.5 mm diameter) in the skull will be made around the planned margin of the implant (as determined by presurgical high-resolution MRI). The implant will be secured to the skull using ceramic skull screws inserted into the holes and acrylic covering the screws. The head post will then be attached to the left side of the skull using acrylic, and the recording chamber will be positioned over the right hemisphere and secured to the implant with screws that slot into plastic nuts in the implant. We will then suture (3–0) the incision to bring skin together around the implant margin. In the subsequent NHP craniotomy surgery, up to five small (2 mm) craniotomies will be made within the recording chamber above the target cortical sites, the precise locations of which will be determined by presurgical high-resolution MRI. 3D printed parts (modified from [41]) will be used to hold the probes. The 3D printed connector will be secured to the skull with dental acrylic at each craniotomy, then the 3D printed probe holder will be inserted into the connector using a customized guide and a micromanipulator system, and secured with small screws. This will seal the craniotomy site and allow the probe to be held in place across sessions under the recording chamber. After surgeries, animals will receive an antibiotic (cefazolin, up to 30 mg/kg IM) and an analgesic agent (buprenorphine, 0.01–0.02 mg/kg IM, every 8–12 hours for at least the first 48 post-operative hours).

When NHP experiments have been completed, animals return to the general population of the animal colony. However, should a case arise in which euthanasia is necessary due to poor health, euthanasia will be carried out with an overdose of sodium pentobarbital (or equivalent) as approved by a clinical veterinarian and according to the current recommendations of the Panel on Euthanasia of the American Veterinary Medical Association.

In mice, we will perform either a whole-hemisphere craniotomy (WHC) that replaces the skull and dura mater above the recording sites with a custom-built implant [42] or small craniotomies over the target locations. For the WHC route, the head post and craniotomy surgeries will be performed simultaneously, prior to behavioral training. For small craniotomies, the head post surgery will be performed before behavioral training while the craniotomy surgery will be performed the day before the recording. Prior to any surgical procedure, mice will be administered subcutaneous injections of sustained release buprenorphine (1 mg/kg) for analgesia, dexamethasone (0.2 mg/kg) for reduction of swelling, and bupivacaine (2 mg/kg), for local anesthesia.

All mice surgeries will start with preparing the scalp through hair removal and antiseptic cleansing with 10% povidone-iodine solution (Betadine, U.S.A.) and 70% ethanol. Then, the scalp skin will be incised and removed to expose the skull. For the small craniotomy head post surgery, a metal head post will be adhered to the skull using dental acrylic, and vetbond will be used to close up the scalp skin. For the subsequent craniotomies, the skin will be reopened and small (~1 mm) craniotomies will be made using a drill. The locations will be determined using Pinpoint [43] and stereotaxic coordinates. For the WHC surgery, the mouse’s head will then be leveled and an outline of the WHC will be made on the left hemisphere using Dumont #5/45 Forceps (Fine Science Tools, U.S.A.). A custom-built headframe will then be adhered to the skull using dental acrylic. Next, the skull will be drilled along the WHC outline and subsequently removed. The dura mater will then be removed using a Bonn Micro Probe (Fine Science Tools, U.S.A.) and scissors (McPherson- Vannas Scissors, 8 cm, 45-degree angle, World Precision Instruments, USA). The exposed brain will then be covered with the custom-built implant which contains holes centered at the intended recording sites (bregma coordinates: anterior/posterior (AP), medial/lateral (ML): ORB: AP + 2.35, ML −1.05; ACA: + 1.1, −0.8; PTLp: −2.25, −1.8; AUD: −2.7, −4.05; VIS: −3.6, −2.3) that are covered by a removable protective silicon layer during behavioral training. The implant will be secured to the skull edges using Loctite 4305 (Loctite, U.S.A) and dental acrylic. Mice will receive a subcutaneous injection of an antibiotic, enrofloxacin (Baytril; 5 mg/kg), for 3 days as part of their post-operative care. At least one day before an experiment, the silicon layer of this implant will be removed to allow for probe insertion into the brain at the defined recording sites. This layer will be replaced with Kwiksil (World Precision Instruments) or Dura-Gel (Dowsil 3–4680).

When mouse experiments have been completed, euthanasia will be performed by infusion of ketamine (300 mg/kg) and dexmedetomidine (3 mg/kg) or prolonged exposure to CO_2_. Both methods are consistent with the recommendations of the American Veterinary Medical Association (AVMA) Guidelines on Euthanasia.

### Electrophysiology experiments

We will use Neuropixels probes to record spiking activity and LFPs simultaneously from two PFC areas, two sensory areas and one parietal area in NHPs and mice during the behavioral task across multiple days. As detailed above, these areas are areas 45 and 46, pITC, mITC and area 7a in NHPs and ACA, ORB, VISp, AUDp and PTLp in mice. The feasibility of simultaneous recordings with Neuropixels has been shown in both mice [42] and NHPs [34,44]. Brain areas will be targeted using high-resolution structural MRI for NHPs (3D T1-weighted images, using an inversion-recovery prepared gradient echo sequence) and, for mice, by using stereotaxic coordinates and manipulator angle verified with sensory response properties and/or reconstruction of probe trajectories by coating probes with DiI or DiO and using *ex vivo* brain imaging or post-hoc histology. For NHPs, ITC face patches will be identified by anatomical “bumps” on the lateral bank of the superior temporal sulcus [45]. Probes in NHPs will be inserted in the right hemisphere as there is a possible right-hemispheric bias for face processing [46,47], whereas probes in mice will be inserted in the left hemisphere to leverage existing surgical and electrophysiological infrastructure.

For NHPs, we will semi-chronically implant (up to five of) the NHP version of the Neuropixels probes with probes attached to customized 3D-printed holders affixed to the skull (10 mm probes for frontal sites, and 45 mm probes for posterior sites) and, for mice, we will acutely insert (up to six) Neuropixels 1.0 probes on the day of the recording using Pinpoint software to guide locations.

### PFC perturbation experiments

Activity in the PFC will be perturbed in a subset of recording experiments, as a causal test of GNWT versus IIT. According to GNWT, this should influence “ignition”, the phasic increase in neuronal activity that is necessary for conscious experience; whereas for IIT, conscious experience does not depend critically on such phasic activity in the frontal cortical areas.

Manipulation trials will be randomly interleaved with no-stimulation trials. PFC activity manipulation will occur from 0–0.5 s after stimulus onset in NHPs and mice, for irrelevant stimuli. Manipulation experiments in will only use one stimulus duration (0.5 s), in order to have a sufficient number of trials for analysis. If the causal manipulation at manipulation onset or offset induces consistent inappropriate behavioral responses (saccades/ licking on irrelevant trials), then we will extend the manipulation duration from 0.1 s before sensory stimulus onset to the end of the behavioral response window. We will also perform control trials in mice in which manipulation is performed outside of the sensory stimulation epoch and with wild-type mice that do not express channelrhodopsin. For NHPs, control trials will also be performed where electrical stimulation occurs during central fixation only.

Manipulation of PFC activity will be achieved by electrical stimulation (NHPs) and optogenetic silencing (mice) of the PFC on a trial-to-trial basis. For NHPs, we will replace Neuropixels in PFC (areas 45/46) with linear electrode arrays (e.g., 24-contacts, 200 µm spacing between contacts) for stimulation across cortical layers (stimulating electrodes held by the customized holders), after completing simultaneous recordings from PFC and posterior cortical sites. We will use high-frequency stimulation (e.g., 100 Hz, 200 µA, 400 µs biphasic pulses, simultaneously applied across 16 stimulating electrode contacts, to perturb “ignition”). In mice, we will use injections of a virus carrying the Cre-dependent red-shifted opsin ChrimsonR [36] or a similar red-shifted excitatory opsin into the ACA and ORB regions of a pan-inhibitory reporter line (Slc32a1-Cre a.k.a. Vgat-ires-Cre). Red light is less scattered by brain tissue, thus can reach deeper regions such as ORB with higher power. Photoactivating opsin-expressing inhibitory neurons effectively silences cortical activity and is a well-established technique used in our labs and others [37,48]. We will use a large diameter fiber-coupled light-emitting diode to provide broad-field PFC illumination, or a focused scanning laser to deliver light to the regions of interest, bilaterally. We will validate the perturbational effect with electrophysiological recordings in pilot experiments prior to the main experiment. If methodological issues prevent the above method, we will use the VGAT-ChR2 mouse line (Vgat-ires-Cre x Ai32), in which channelrhodopsin is expressed in all GABAergic cortical neurons. Photoactivating the cells in this line effectively silences cortex activity and is a well-established technique used in our labs and others [37,48].

## Analysis plan

### Neuronal data pre-processing

We will acquire high-pass-filtered (>500 Hz) signals sampled at 30 kHz (the action potential band), and low-pass filtered signals (<1 kHz) sampled at 2.5 kHz (the local field potential band) using the Open Ephys GUI [49]. The LFP band data will be pre-processed in MATLAB or Python to remove noisy channels or probes. The raw action potential (AP) band data will be pre-processed and then spike-sorted by a standardized spike-sorting pipeline that uses Kilosort via SpikeInterface [50]. Spike sorting will be followed by multi-step curation, including assessment of drift metrics, refractory period violations, and waveform properties. We will apply the same set of quality metrics and curation procedures across all datasets and laboratories to ensure consistency and reproducibility. Based on our previous experience we expect to record from 50–200 single units in the cortex per probe per session.

All spike data, LFP data, and behavioral metadata will be packaged into the Neurodata Without Borders [51] 2.0 data standard for sharing between groups within the project and for ultimate public release. Spike rate, LFP power and current source density (to localize cells/LFPs to cortical layers) analyses will also be performed using MATLAB or Python-based software [34,52].

### Decoding analysis

We will perform decoding analyses using openly shared tools [52,53]. Briefly, we will calculate the number of spikes, sorted at the level of individual units, in each time window (dependent on the question; see Analysis plan for predictions below) across the trial [54]. Only ‘correct’ trials will be included (i.e., correct response to target stimulus and no response to non-target and irrelevant stimuli). For mice, we will only include trials when the mice were actively engaged in the task, defined as at least an 80% response rate to target trials in the preceding and following two minutes. For NHPs, we will only include trials during stable task performance with an overall accuracy of greater than 75% per session. We will then use a classifier, e.g., a support vector machine (SVM), scikit- learn package sklearn.svm.LinearSVC with ten-fold cross-validation at each bin independently (i.e., using spike counts for all cells in one session in each bin as input features, and classified stimulus label at each bin as output) [55].

When content-specific sensory region decoding is involved in the analysis, the recording data will be from the sensory region that is associated with the sensory content in the relevant trial. For example, if the stimulus being decoded is faces (NHPs) or visual gratings (mice), then we will use pITC (including PL), mITC (including ML) and VISp as the content-specific sensory regions, respectively.

For NHPs, we will use neural activity to decode category (face vs. object) both within-condition (i.e., train and test with cross-validation for non-target relevant faces vs. non-target relevant objects, and separately for irrelevant faces vs. irrelevant objects) and across-condition (e.g., train on non-target relevant faces vs. non-target relevant objects, and test on irrelevant stimuli). Across-condition decoding will be used for Question 1 and within-condition for Question 2 (see Analysis plan for predictions below). We will also decode stimulus orientation within category, a variable that was never reinforced, for non-target relevant and irrelevant stimuli combined (to yield sufficient trial numbers). For theories to pass questions involving decoding, decoding of both category and orientation (in at least one category) is required, reflecting the multidimensional character of consciousness.

Since each mouse only experiences one modality as relevant, for mice we will focus on the irrelevant stimuli and decode their identity within-condition (i.e., train and test with cross-validation on irrelevant tone 1 vs. irrelevant tone 2 in a visual-trained mouse) for all decoding-related questions. This analysis is comparable to orientation decoding in NHPs, since the irrelevant stimuli were never reinforced, and it eliminates potential confounds from movement or reinforcement-related neural activity.

This decoding analysis is crucial, as both Integrated Information Theory (IIT) and Global Neuronal Workspace (GNW) posit that every consciously experienced content (here a stimulus feature such as orientation) should be decodable from activity in brain areas relevant to consciousness. Successful decoding would thus provide empirical support for the presence of orientation information within the neural substrate of conscious perception.

### Functional connectivity analysis

We will use the pairwise phase consistency metric to assess functional connectivity between brain areas’ spikes and LFPs. Content-specific spike-LFP pairwise phase consistency will be calculated for non-target and irrelevant stimuli. The pairwise phase consistency metric is unbiased by the number of trials or spikes [56,57]. For each neuron, we will calculate the LFP phase relative to individual spikes, using the Fourier transform of the LFP (Hanning windowed). We will then calculate the pairwise phase consistency using the FieldTrip toolbox [58]. When calculating the pairwise phase consistency from channels on the same probe, we will exclude LFP channels within 40 microns of the best channel of the sorted unit. To test the predictions of IIT, we will apply pairwise phase consistency analyses to neurons and LFPs significantly activated (based on a t test) in response to the stimuli, i.e., increased spike rate for neurons or increased gamma power for LFPs, in the window between 250 ms post-stimulus onset to stimulus offset for NHPs and between 150 ms post-stimulus onset to stimulus offset for mice, compared to baseline (0–250 ms pre-stimulus onset). To test the predictions of GNWT, we will apply pairwise phase consistency analyses between significantly responsive neurons/LFPs (in the window 250–500 ms post-stimulus onset for NHPs and 150–400 ms post-stimulus onset in mice, compared to the baseline 0–250 ms pre-stimulus onset) and all other LFPs/neurons located in putative GNWT brain areas, because GNWT predicts that the early local sensory response is broadcast during ignition into the global workspace and made available to its components, whether or not each of them adds additional processing.

## Statistical and sampling plan

### Bayes factor analysis

We will use Bayesian t tests and a (half) Cauchy distribution (width, r = 0.707) to calculate the Bayes factors (BFs) which will be used to interpret evidence for each theory. For each question, we will aim to continue data collection until the BF is at least 10X in favor for either of our alternative/null hypotheses (and at least nine recording sessions from each macaque, and nine recording sessions from each region of interest in mice, based on frequentist power analysis below). However, we will enforce a sample size limitation of 15 recording sessions in each NHP and, for mice, 4 recordings per area per mouse, with a maximum of 20 mice. These sample sizes take into account factors such as the viability of probes in chronic recordings in NHPs and the effects of multiple probe penetrations in acute recordings in mice. The theory interpretations of each question are still feasible even if these maximal sample sizes yield a lower BF than 10 (see Table 1), i.e., BFs from 3 to 10 will be considered as moderate evidence, and BFs larger than 10 considered as strong evidence.

### Frequentist statistical analysis

We will also report frequentist statistics, to complement the information provided by the BFs (although evidence for each theory will be based on BFs). We will perform at least nine recording sessions from each macaque (two total), and nine recording sessions from each region of interest in mice. Based on our pilot recordings of ≥50 units per region of interest per session, we will exceed robust sample sizes for decoding, estimated from our pilot analyses, and published sample sizes yielding robust functional connectivity results from pairwise phase consistency analyses [55–57]. All neurons and correct trials will be included in our analyses. Our sampling plan is based on the power analysis of pilot data from Neuropixels recordings in the visual (VISp) and/or auditory (AUDp/AUDpo/AUDd) cortex during the mouse behavioral task (Fig 1 in S1 Supporting Information in S1 File). We trained an SVM classifier to decode the identity of the task irrelevant stimulus using mean spike counts measured from simultaneously recorded neurons (Fig 2 in S1 Supporting Information in S1 File). This classifier accurately decoded stimulus identity in both the visual-relevant group (N = 3 mice) and the auditory-relevant group (N = 4 mice). We computed the pooled standard deviation of classifier performance (using 25 neurons and 30 trials/condition, when decoder performance saturated) and used this to estimate the number of samples needed given an alpha value of 0.05 and power of 0.95. Because we seek relatively large effect sizes, we set a threshold for decoding performance at 0.7, which equates to an effect size of 1.93 and a required sample size of 8.09 at 0.95 power (rounded up to nine recording sessions). See S1 Supporting Information for full details in S1 File.

## Discussion

### Analysis plan for predictions

#### Question 1a: Where is conscious content represented?.

***GNWT prediction:*** Stimuli are decodable from PFC and PPC.

***IIT prediction:*** Stimuli are maximally decodable from PPC and/or sensory regions.

To address the theory predictions regarding the location of conscious content in the brain, we will assess the average decoding performance in the PFC, PPC, and content-dependent sensory regions during both the stimulus onset window (250–500 ms post-stimulus onset for NHPs and 150–400 ms post-stimulus onset for mice) and the stimulus duration window (250 ms post-stimulus onset – stimulus offset for NHPs and 150 ms post-stimulus onset – stimulus offset for mice). These time windows were chosen to reflect the likely time period in which conscious processing of the stimuli in the predicted cortical areas would occur. Subsequently, the mouse time window is shifted earlier than the NHP window, due toknown faster processing speeds of neuronal signals to the cortex [31]. Output labels will be randomly permuted (1,000 times) for each time window, and actual and permuted decoding accuracies will be compared. A Bayesian t test will also be applied for each time window, comparing evidence for greater than chance decoding using a half Cauchy prior (width, r = 0.707) versus chance decoding with a point nil.

**Fig 3:**
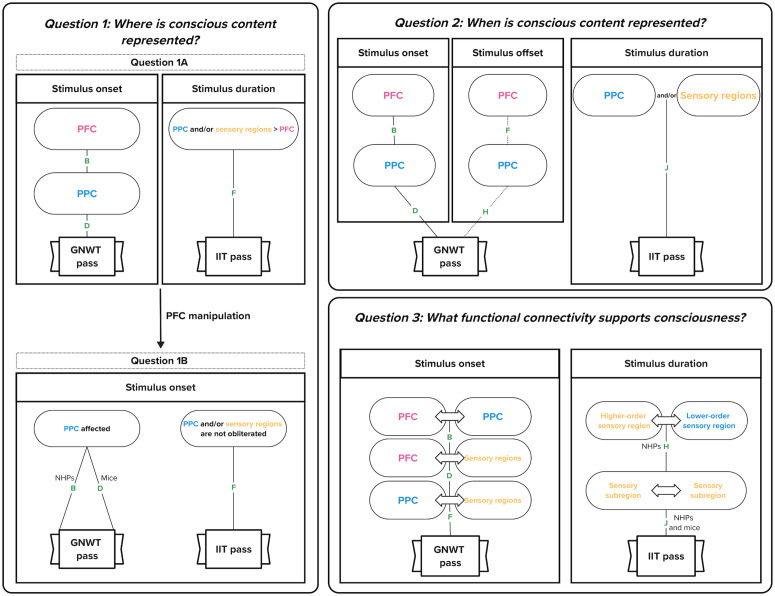
Schematic of the analysis plan in relation to the theory-specific predictions. This figure displays the decoding, activation and connectivity-based tests designed to assess the predictions from the Global Neuronal Workspace Theory (GNWT) and Integrated Information Theory (IIT) across three key questions. Bayesian model comparison is used across all analyses. Bayes Factors (BF) > 10 are interpreted as strong evidence for the alternative hypothesis (H_1_), while BF < 1/10 supports the null hypothesis (H_0_). Letters next to each arrow (A–J) correspond to hypothesis outcomes listed in Table 1. Pass criteria for each theory are indicated by framed “GNWT pass” or “IIT pass” labels within each panel. Question 1: Where is conscious content represented? Question 1A (top left): Decoding performance is compared across prefrontal cortex (PFC), posterior parietal cortex (PPC), and sensory regions. GNWT predicts above-chance decoding in both PFC (B) and PPC (D) during the stimulus onset window (left panel), while IIT predicts stronger decoding in PPC and/or sensory regions than in PFC (F) during the stimulus duration window (right panel). Question 1B (bottom left): The effect of PFC manipulation on decoding in PPC and/or sensory regions is assessed. GNWT predicts a change in PPC decoding during the stimulus onset window (D for mice, B for NHPs). IIT predicts above chance decoding will persist in PPC (A and C) and/or sensory regions (E and G) during the stimulus onset window. Question 2: When is conscious content represented? Decoding generalization is tested across time using a sliding 250 ms window. GNWT predicts above-chance generalization in both PFC (B) and PPC (D) during the stimulus onset window (left subpanel). It also predicts a second ignition signal at stimulus offset (center subpanel), shown by significantly increased spiking compared to baseline (as decoding is not possible after stimulus offset) (F, H). IIT predicts sustained decoding generalization throughout the stimulus duration period in PPC and/or sensory regions (J; right subpanel). Question 3: What functional connectivity supports consciousness? Pairwise phase consistency between brain regions is measured to evaluate changes in functional connectivity. GNWT predicts increased connectivity from pre-stimulus to stimulus onset between PFC and PPC (B), and between PFC/ PPC and sensory regions (D, F). IIT predicts increased connectivity during the stimulus duration period within and between sensory regions (H, J). Dashed lines represent weak theoretical commitment.

To test GNWT’s prediction that conscious content is encoded in the PFC and PPC, we will test whether average decoding performance is greater than chance in PFC and in PPC in the stimulus onset time window. A BF greater than 10 will be interpreted as strong evidence for alternative hypothesis (H_1_), that performance is greater than chance (Table 1: PFC: B; PPC: D), while a BF less than 1/10 will be taken as strong evidence for null hypothesis (H_0_), that performance is equal to chance (Table 1: PFC: A; PPC: C). A pass for GNWT requires evidence that decoder performance is above chance in both PFC (Fig 3 and Table 1: B) **and** PPC (Fig 3 and Table 1: D).

To test IIT’s alternative prediction that conscious content is instead maximally encoded in the PPC and/or sensory regions than in the PFC, we will compare the average decoding performance between these regions, within the stimulus duration window. A BF greater than 10 will be interpreted as strong evidence for H_1_, that performance is greater in PPC and/or sensory regions (Table 1: F), while a BF less than 1/10 will be taken as strong evidence for H_0_, that performance is equal between regions (Table 1: E). A pass for IIT requires evidence that decoder performance is higher in PPC and/or sensory regions than in the PFC (Fig 3 and Table 1: F).

#### Question 1b: Is PFC required for consciousness?.

***GNWT prediction:*** Decoding from PPC should be modified (increased or decreased) by PFC manipulation, due to interference with ignition, while decoding from sensory areas (visual or auditory cortices) should be preserved in the early time-window before ignition.

***IIT prediction:*** Decoding from PPC and/or sensory regions is not obliterated by PFC manipulation.

In order to test the theories’ differing predictions about whether the PFC is a required brain area for consciousness, we will assess whether the average decoding performance is different during PFC manipulation trials (cf. no manipulation trials) in the PPC (GNWT) and PPC/sensory regions (IIT), within the stimulus onset time window.

To address GNWT’s prediction that the PFC is required for consciousness due to ignition, we will test whether the average decoding performance in PPC is affected by PFC manipulation in the stimulus onset window. For each comparison, a BF less than 1/10 will be taken as strong evidence for the H_0_, that there is no difference (Table 1: H_0_ vs. H_1_: A; H_0_ vs. H_2_: C). A BF greater than 10 will be interpreted as strong evidence for either H_1_, that PPC performance is altered with PFC manipulation (Table 1: B) or H_2_, that PPC is lower with PFC manipulation (Table 1: D). GNWT predicts that decoding performance in the PPC will be affected by PFC manipulation, specifically, performance should be lower for mice and either higher or lower for NHPs, based on higher confidence in the inhibitory effect of the PFC manipulation in mice vs. NHPs [37,48,59,60]. Thus, a GNWT pass requires that decoder performance in PPC after PFC manipulation is lower in mice (Fig 3 and Table 1: D) **and** different in NHPs (Fig 3 and Table 1: B **or** D).

To test IIT’s prediction that PFC is not required for consciousness, we will also assess whether average decoding performance in the PPC and/or sensory regions is not obliterated by PFC manipulation in the stimulus onset window. A BF greater than 10 will be interpreted as strong evidence for H_1_, that PPC and/or sensory region decoding performance is greater than chance with PFC manipulation (Table 1: F). A BF less than 1/10 will be taken as evidence for H_0_, that decoding performance is equal to chance after manipulation (Table 1: E). To pass, IIT requires performance to be greater than chance with PFC manipulation for PPC and/or sensory regions (Fig 3 and Table 1: F).

#### Question 2: When is conscious content represented?.

***GNWT prediction:*** Decoding generalizes in PFC and PPC during ignition, and continues as long as the stimulus is consciously perceived. As it is not clear whether the subject continues to consciously see the stimulus at later times, as well as notices the offset as neither is task-relevant, GNWT does not make any strong prediction about stimulus offset. A bimodal distribution of cross-condition decodability (high versus chance decodability) might be observed reflecting this variability of conscious access to stimulus offset.

***IIT prediction:*** Decoding in PPC and/or sensory regions generalizes across stimulus duration.

To assess the theories’ predictions regarding the timing of the neuronal processing of conscious content, we will look at whether decoders trained on multiple time bins using a sliding window approach are able to generalize to the theory-specified time windows: the stimulus onset window (GNWT) and stimulus duration window (IIT). The average cross-temporal decoder generalization will be computed by training the decoder in a 250 ms window moving every 100 ms, from 250 ms pre-stimulus onset to 250 ms post-stimulus offset, and testing each time window’s decoder on every other time window’s data. Output labels will be randomly permuted (1,000 times) for each time window, and actual and permuted decoding accuracies will be compared at each window, with Holm’s correction applied to account for multiple comparisons. A Bayesian t test will also be applied at each time window, comparing evidence for greater than chance decoding using a half Cauchy prior (width, r = 0.707) versus chance decoding with a point nil.

To test GNWT’s prediction that conscious processing is neuronally represented by an ignition signal just after stimulus onset in PFC and PPC, we will assess whether average decoding performance generalization is above chance level, in both PFC and PPC, in the stimulus onset window (250–500 ms after stimulus onset for NHPs, and 150–400 ms after stimulus onset for mice). A BF greater than 10 will be interpreted as strong evidence for H_1_, that decoding generalization is higher than chance (Table 1: PFC: B; PPC: D), and a BF less than 1/10 will be taken as strong evidence for H_0_, that decoding generalization is equal to chance (Table 1: PFC: A; PPC: C).

To test GNWT’s prediction that the ignition signal should also occur just after stimulus offset, we will assess whether spike rate in PFC and PPC in the stimulus offset window (250–500 ms after stimulus offset for NHPs, and 150–400 ms after stimulus offset for mice) is greater than the baseline spike rate (0–250 ms before stimulus onset) using a t test and Bayesian t test. The blank screen after stimulus offset precludes the use of decoding in this period. A BF greater than 10 will be interpreted as strong evidence for H_1_, that spike rate at stimulus offset is greater than at baseline (Table 1: PFC: F; PPC: H), and a BF less than 1/10 will be taken as evidence for H_0_, that spike rate at stimulus offset is equal to that at baseline (Table 1: PFC: E; PPC: G).

However, to account for the possibility that on some trials animals may not be consciously aware of the stimulus towards the end of the presentation window and thus, would not generate the offset ignition signal corresponding to the new experience, we will first assess this possibility and then attempt to filter these cases out (but see controls – including surprise memory task – for similar human task which suggest subjects are aware of the stimulus across its duration for both relevant and irrelevant stimuli [6]). To test this, we will first measure the average variance in spike rate in the stimulus onset window and compare it to the stimulus offset window, in both PFC and PPC cells, using an F test (α = 0.05). Significantly higher variance post-stimulus offset may suggest bimodal responses. If variance is not significantly different, we will run the spike rate analysis to test ignition at stimulus offset on all trials. If variance is significantly different in the stimulus offset window, we will extract the spike rates in this window and test for a bimodal distribution using the dip test (α = 0.05). If there is no bimodality, we will run the spike rate analysis to test ignition at stimulus offset on all trials. If there is bimodality, we will use Gaussian mixture models (assigning trials to the Gaussian distribution with highest probability) to split the trials into the low and high spiking groups (which may reflect perceptual differences between the groups), and run the spike rate analysis to test ignition at stimulus offset on the high group.

For GNWT to pass this question, generalization is required to be greater than chance in the stimulus onset window in both PFC (Fig 3 and Table 1: B) **and** PPC (Fig 3 and Table 1: D). Due to the possibility that on some trials animals may not be consciously aware of the stimulus towards the end of the presentation window and thus, would not generate the offset ignition signal corresponding to the new experience, there is only a weak theoretical commitment from GNWT to the requirement that the spike rate be greater than baseline in the stimulus *offset* window in both PFC (Fig 3 and Table 1: F) **and** PPC (Fig 3 and Table 1: H).

To test IIT’s prediction that conscious content will be neuronally represented across the duration of stimulus presentation, we will assess whether average decoding performance generalization is above chance level in the PPC and/or sensory regions, across the stimulus duration time window. A BF greater than 10 will be interpreted as strong evidence for H_1_, that decoding generalization is higher than chance (Table 1: J), and a BF less than 1/10 will be taken as strong evidence for H_0_, that decoding generalization is equal to chance (Table 1: I). For a pass, IIT requires generalization across stimulus duration to be greater than chance (Fig 3 and Table 1: J).

#### Question 3: What functional connectivity supports consciousness?.

***GNWT prediction:*** Increased connectivity between PFC and PPC and sensory regions during ignition post-stimulus onset and offset.

***IIT prediction:*** Maintained content-specific connectivity within and between sensory regions, across stimulus duration.

To test the theories’ diverging predictions regarding the functional connectivity of the neuronal representation of conscious content, we will look at whether there is an increase in the average pairwise phase consistency between PFC and PPC (for GNWT), between PFC and sensory regions (for GNWT), between PPC with sensory regions (for GNWT), between sensory regions (only in NHPs for IIT) and within sensory regions (for IIT), from the baseline pre-stimulus onset window (0–250 ms pre-stimulus onset) to the stimulus onset window (250–500 ms post-stimulus onset for NHPs and 150–400 ms post-stimulus onset for mice; for GNWT) and stimulus duration window (for IIT). To calculate this, we will first find the spike-LFP pairwise phase consistency [56,57] for each area pair being tested, then, we will compare the consistency at pre-stimulus onset with both the consistency at the two tested time windows. We will use t tests and Bayesian t tests.

To test GNWT’s prediction that connectivity between the PFC and PPC with sensory brain regions increases after stimulus presentation, we will assess whether the difference in pairwise phase consistency is greater than baseline in the stimulus onset window in the PFC and PPC pair, the PFC and sensory regions pair, and the PPC and sensory regions pair. A BF greater than 10 will be interpreted as strong evidence for H_1_, that the difference in pairwise phase consistency is greater than baseline (Table 1: PFC/PPC pair: B; PFC and sensory regions pair: D; PPC and sensory regions pair: F), while a BF less than 1/10 will be taken as strong evidence for H_0_, that pairwise phase consistency is equal to baseline (Table 1: PFC/PPC pair: A; PFC and sensory regions pair: C; PPC and sensory regions pair: E). To pass, GNWT requires the pairwise phase consistency increase to be greater than baseline in the stimulus onset window in the PFC and PPC pair (Fig 3 and Table 1: B) **and** the PFC and sensory regions pair (Fig 3 and Table 1: D) **and** the PPC and sensory regions pair (Fig 3 and Table 1: F).

To test IIT’s prediction that connectivity within the activated posterior brain regions is increased in the duration of stimulus presentation, we will look at whether the difference in pairwise phase consistency is greater than baseline in the stimulus duration window within and between the activated neurons/LFPs in sensory regions. A BF greater than 10 will be interpreted as strong evidence for H_1_, that the difference in consistency is greater than baseline (Table 1: higher-order and lower-order sensory regions pair in NHPs: H; within sensory regions pair: J), while a BF less than 1/10 will be taken as strong evidence for H_0_, that pairwise phase consistency is equal to baseline (Table 1: higher-order and lower-order sensory regions pair: G; within sensory regions pair: I). To pass, IIT requires the pairwise phase consistency increase to be greater than baseline in the stimulus duration window in the higher-order and lower-order sensory region pairs (Fig 3 and Table 1: H) **and** within sensory regions (Fig 3 and Table 1: J).

## Supporting information

S1 File Pilot data. (DOCX)
