# Supplementary material for: Protocol for testing global neuronal workspace and integrated information theories of consciousness in non-human primates and mice
Source: PLoS One. 2026 Feb 25;21(2):e0342770. doi: 10.1371/journal.pone.0342770 (PMC12935206; doi:10.1371/journal.pone.0342770)
Supplement: S1 File — (DOCX) [file pone.0342770.s001.docx]

**Supporting information**

**Pilot data**

**Mouse behavior pilot**

To establish the feasibility of the mouse behavioral task, we performed a set of pilot experiments. We trained seven mice to perform the “visual-relevant” task and a set of six mice to perform the “auditory- relevant” task. Supplementary Figure 1 shows that both groups of mice were successfully able to learn the behavioral task. The visual-relevant mice responded with a high hit rate for the visual target stimuli, and only responded at very low rates for the task-relevant visual distractor and both task-irrelevant auditory stimuli (Supplementary Fig. 1a,b). Likewise, the auditory-relevant mice responded with high hit rates to the task-relevant auditory target, and not the auditory distractor or task irrelevant visual stimuli (Supplementary Fig. 1c). These results indicate that mice can successfully learn and perform the behavioral task to be used in this study.


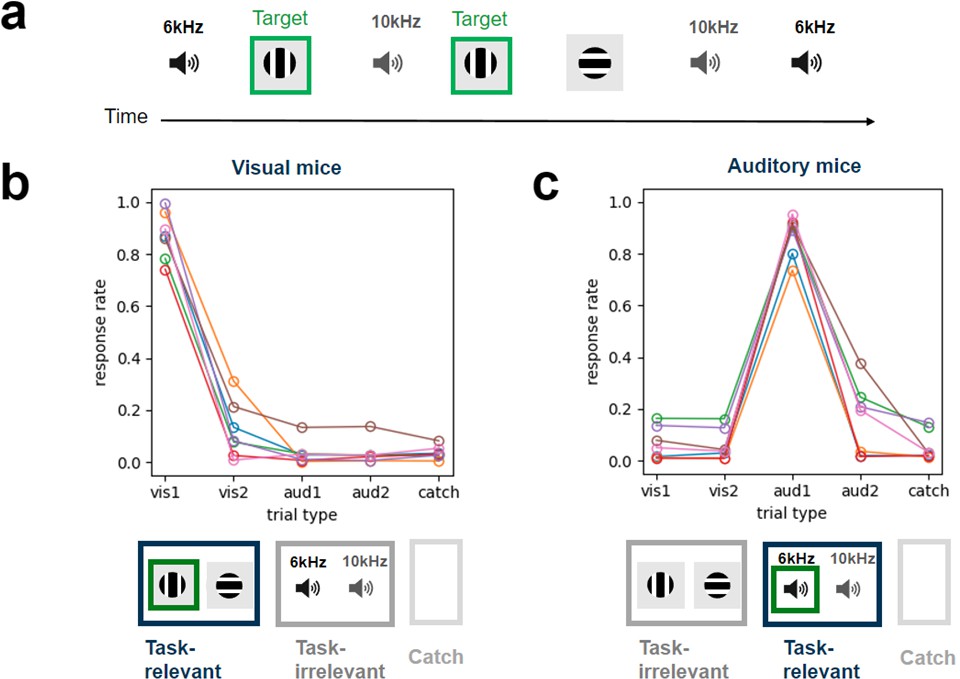


**Supplementary Figure 1. Pilot results demonstrating feasibility of the mouse behavioral task. a,** Schematic of the stimuli sequence presented to the mice. The “visual-relevant” task is shown here. Note all stimuli are randomly interleaved. On 10% of trials, no stimulus was presented; these “catch trials” allowed assessment of the mouse’s guess rate in the absence of a stimulus. **b,** Performance of six mice trained on the “visual-relevant” task. The mice responded with high rates to task-relevant target stimuli (vis1), but not to distractors or catch trials. **c,** Performance of six mice trained on the “auditory-relevant” task.

**Neuropixels recordings in mice**

We collected a pilot dataset in which we targeted Neuropixels probes to the visual (VISp) and/or auditory cortex (AUDp/AUDpo/AUDd) during the mouse behavioral task. We sought to determine how the

number of units recorded and the number of trials performed would impact the decodability of stimulus information content in the cortex. We also used these pilot experiments to perform a power analysis to estimate sample sizes needed to test the decoding predictions in Table 2.


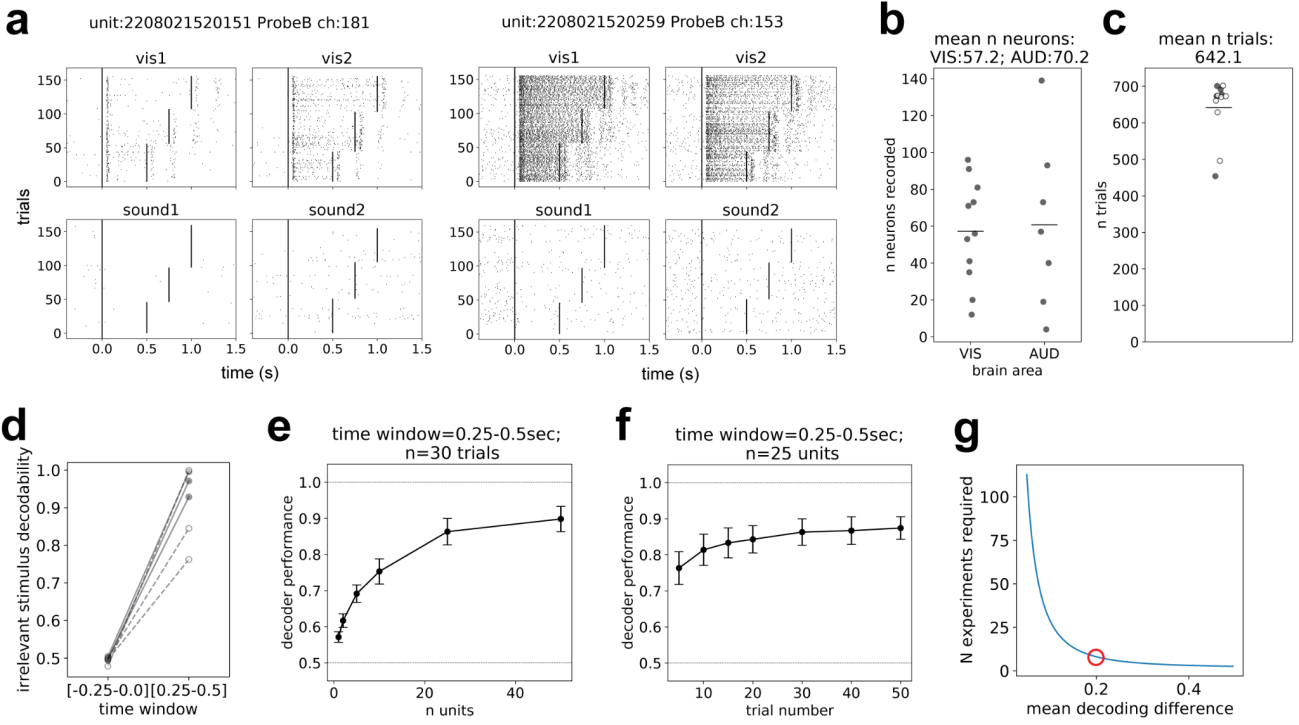


**Supplementary Figure 2. Pilot Neuropixels recordings and power analysis to estimate required sample size. a,** Raster plots of spiking responses for two example neurons (left, neuron 1; right, neuron 2) recorded in visual cortex. Each plot shows responses to one of the four stimuli used: vis1 (vertical grating), vis2 (horizontal grating), sound1 (6 kHz tone), sound2 (10 kHz tone). For each stimulus type, 3 stimulus durations were tested including 500 ms, 750 ms, and 1000 ms. The rasters are grouped by stimulus duration and the start and end of the stimulus presentation is indicated by a black line. This example mouse is an “auditory mouse” which had been trained to respond to auditory targets but to ignore visual stimuli. **b,** Number of neurons recorded per probe from either the visual cortex or auditory cortex per experiment. Each point corresponds to one experiment. **c,** Total number of trials performed by the mice in each experimental session. Mean (642 trials) is shown within the horizontal bar. **d,** Decoder performance for task irrelevant stimuli. Performance was high when training a classifier on the time window between 250-500 ms after stimulus onset but was at chance level (50%) when the classifier was trained on spikes prior to stimulus presentation. **e,** Decoder performance (mean+/-SEM) as a function of the number of neurons used in the decoding. **f,** Decoder performance (mean+/-SEM) as a function of the number of trials used in the decoding. **g,** Number of samples (i.e. probe recordings) from each area that was required to detect an effect size expressed as the difference in mean decodability from chance. We chose an effect size (mean difference of 0.2) to be the threshold for passing/failing decodability predictions (red circle). This corresponds to a sample size of 8.1 recordings for each area.

**Power analysis**

We focused our analysis specifically on task-irrelevant stimuli (e.g. visual stimuli for auditory trained mice, and auditory stimuli for visually trained mice), since these stimuli are the substrate of the decoding predictions. As expected, we found that single units in the sensory cortex responded robustly to task-

irrelevant stimuli. For instance, in auditory-relevant mice, we found neurons in the visual cortex that were reliably responsive to the task-irrelevant visual stimuli (Supplementary Fig. 2a). In the experiments where we successfully hit the target areas (verified post-hoc with histology), we recorded from ~55-70 units in visual and auditory cortex (Supplementary Fig. 2b), while the mouse performed >600 trials on average (Supplementary Fig. 2c). Overall, each stimulus condition was repeated ~50 times per experimental session (4 stimuli × 3 durations = 12 trial types + 10% catch trials; (642 trials - 642 × 0.1 catch trials) / 12 trial types = 48.2 trials per session).

We trained an SVM classifier with a linear kernel to decode the identity of the task irrelevant stimulus using mean spike counts measured from simultaneously recorded neurons. In our first analysis we used all neurons recorded in each session. In both the auditory-relevant group (N = 4 mice) and the visual- relevant group (N = 3 mice), we could accurately decode the identity of the task irrelevant stimuli (Supplementary Fig. 2d). To estimate the minimum number of trials and units required for the analysis, the classifier was trained including different subsets of units or trials, then training was repeated 100 times for each combination of number of units and trials to obtain an estimate of how decoder accuracy varies. 50% of trials were held out as the test dataset. We found that decoder performance saturated at approximately 25 units (Supplementary Fig. 2e) and 30 trials (Supplementary Fig. 2f), well within the average number of units (47.3) and trials (48.2) per condition from pilot experiments.

We next used these results to perform a power analysis. We computed the pooled standard deviation of classifier performance using 25 units and 30 trials (SD_pooled = 0.1037), and used this to estimate the number of samples needed per group given an alpha value of 0.05, power of 0.95, and a range of effect sizes based on the difference in means between groups (Supplementary Fig. 2g). For this study we seek relatively large effect sizes and so we set a threshold for decodability performance at 0.7, which equates to an effect size of 1.93 and a required sample size of 8.09 at 95% power.
